# Soft Randomized Machine Learning Procedure for Modeling Dynamic Interaction of Regional Systems

**DOI:** 10.3390/e21040424

**Published:** 2019-04-20

**Authors:** Yuri S. Popkov

**Affiliations:** 1 Federal Research Center “Computer Science and Control” of Russian Academy of Sciences, 119333 Moscow, Russia; popkov@isa.ru; 2 Institute of Control Sciences of Russian Academy of Sciences, 117997 Moscow, Russia; 3 Department of Software Engineering, ORT Braude College, 216100 Karmiel, Israel; 4 Yugra Research Institute for Information Technologies, 628011 Khanty-Mansiysk, Russia

**Keywords:** soft randomization, entropy, entropy operator, migration, immigration, empirical balance, empirical risk

## Abstract

The paper suggests a randomized model for dynamic migratory interaction of regional systems. The locally stationary states of migration flows in the basic and immigration systems are described by corresponding entropy operators. A soft randomization procedure that defines the optimal probability density functions of system parameters and measurement noises is developed. The advantages of soft randomization with approximate empirical data balance conditions are demonstrated, which considerably reduces algorithmic complexity and computational resources demand. An example of migratory interaction modeling and testing is given.

## 1. Introduction

The mutual influence of migratory processes in regional systems is a problem of growing significance in the modern world. The socioeconomic statuses of different regions demonstrate higher heterogeneity in response to rising political and military tension. All these factors cause an abrupt redistribution of migration flows and regional population variations, thereby increasing the cost of regional population maintenance [1,2,3,4]. Therefore, it is important to develop different tools (mathematical models, algorithms, and software) for forecasting the distribution of migration flows with adaptation to their dynamics considering available resources.

The authors of [5] suggested a dynamic entropy model for the migratory interaction of regional systems. In comparison with biological reproduction, migration mobility is a rather fast process [1,6]. Thus, the short-term dynamics of regional population size are described by the locally stationary state of a migratory process [7]. The latter can be simulated under the hypothesis that all migrants have a random and independent spatial distribution over interacting regional systems with given prior probabilities. The mathematical model of a locally stationary state is given by a corresponding entropy operator that maps the space of admissible resources into the space of migratory processes [8].

Mathematical modeling and analysis of interregional migration is considered in numerous publications. First, it seems appropriate to mention the monographs [9,10] that are dedicated to a wide range of interregional migration problems, including mathematical modeling of migration flows. Note that the problem of migration touches upon many aspects of socioeconomic, psychological and political status of the space of migratory movements. Thus, of crucial role is the structural analysis of inter- and intraregional migration flows [4] and motivations that generate them [2,11]. The results of structural and motivational analysis of migratory processes are used for computer simulation. There exist three directions of research in this field, each relying on some system of hypotheses. One of the directions involves the stochastic hypothesis about the origin of migratory motivations [12], which is simulated using agent technologies [13,14]. This direction is adjoined by investigations based on the thermodynamic model of migration flows [3,8]. Of course, the short list above does not exhaust the whole variety of migration studies, merely outlining some topics of research.

This paper studies a stochastic version of the model in [5], in which random parameters and measurement noises are characterized by probability density functions (PDFs). These functions are estimated using retrospective information on the real dynamics of regional population size with “soft” randomized machine learning [15]. The learned model was implemented in the form of computer simulations, i.e., generation of an ensemble of random trajectories with the entropy-optimal PDFs of the model parameters and measurement noises. The resulting ensemble was used for testing of the model and also for short-term forecasting.

The method developed below is illustrated by an example of the randomized modeling and forecasting of the migratory interaction among three EU countries (Germany, France, and Italy—the system GFI) and two countries as sources of immigration (Syria and Libya—the system SL).

## 2. Randomized Model of Migratory Interaction

Consider the dynamic discrete-time model of migratory interaction with shared resource constraints that is presented in [5]. The first sub-model represents migration flows within the system GFI and is described by the dynamic regression equation

(1) $$K\left[\left(s+1\right)h\right]=\left(A-E\right)K\left[sh\right]+F\left(z\left[sh\right]\right),\;\left(K,F\right)\in R^{N},\;s=\bar{0,K-1},$$

where

(2) $$A=h\left(\begin{array}{cccc} 1 & \alpha_{2}a_{21} & \cdots & \alpha_{N}a_{N1}\\ \alpha_{1}a_{12} & 1 & \cdots & \alpha_{N}a_{N2}\\ \cdots & \cdots & \cdots & \cdots \\ \alpha_{1}a_{1N} & \alpha_{2}a_{2N} & \cdots & 1 \end{array}\right),$$

(3) $$E=h\;\mathrm{diag}[\alpha_{n},\;n=\bar{1,N}].$$

In these equations, K[sh] denotes the population distribution in the regional system GFI at a time sh.

At a time sh, the distribution of immigration flows from the regional system SL to the regional system GFI in terms of an entropy operator is modeled by the second sub-model, which can be described by a vector function F(z[sh]) with the components

(4) $$f_{n}\left[sh\right]=h\sum_{j=1}^{M}b_{jn}{\left(z\left[sh\right]\right)}^{c_{jn}},\;n=\bar{1,N},\;s=\bar{0,K-1},$$

The variable *z*, which is the exponential Lagrange multiplier in the entropy-optimal distribution problem of immigration flows, satisfies the equation

(5) $$\sum_{k=1}^{M}\sum_{n=1}^{N}c_{kn}b_{kn}{\left(z\left[sh\right]\right)}^{c_{kn}}=T\left[sh\right],$$

where T[sh] is the amount of a shared resource used by all regions from the system GFI to maintain immigrants.

In this model, the input data are the amounts T[0],T[h],…,T[(K−1)h]; and the output data are the regional population distributions K[0],K[h],⋯,K[(K−1)h].

The dynamic model in Equations (1)–(5) contains the following parameters:

- $\alpha_{n}\in \left[0,1\right],\;n=\bar{1,N},$ as the shares of mobile population in system regions;
- $a_{in}\in \left[0,1\right],\;\left(i,n\right)=\bar{1,N},$ as the prior probabilities of individual migration in the system GFI;
- $b_{kn},\;k=\bar{1,M},\;n=\bar{1,N},$ as the prior probabilities of individual immigration from region *k* of the system SL to region *n* of the system GFI; and
- $c_{kn},\;k=\bar{1,M},\;n=\bar{1,N},$ as the normalized 1 specific generalized cost of immigration maintenance.

Normalization means that $0<c_{kn}<1,\;k=\bar{1,M},\;n=\bar{1,N}$.

The parameters form three groups: mobility, migratory movements within the system GFI, and immigratory movements from the system SL to the system GFI. All these characteristics are specified by the regions of both systems. The dimensionality of the parametric space is reduced using the same approach as in [5]. The whole essence is to assign a relative regional differentiation of all parameters except for the weights $b_{1}$ (mobility) and $b_{2}$ (internal migration) of these groups, which are considered as model variables.

This approach leads to the parametric transformation

(6) $$\begin{array}{c} \alpha_{n}=b_{1}m_{n},\;a_{in}=b_{2}h_{in},\\ \left(i,n\right)=\bar{1,N};\;k=\bar{1,M}, \end{array}$$

where $m_{n}$ and $h_{in}$ are given parameters which characterize the relation of variables.

Then, the dynamic model of migratory interaction in Equations (1)–(5) takes the form

(7) $$K\left[\left(s+1\right)h\right]=\left(b_{1}b_{2}\tilde{A}-b_{1}\tilde{E}\right)K\left[sh\right]+\tilde{F}\left(z\left[sh\right]\right),$$

with the matrix

(8) $$\tilde{A}=h\left(\begin{array}{cccc} 1 & m_{2}h_{21} & \cdots & m_{N}h_{N1}\\ m_{1}h_{12} & 1 & \cdots & m_{N}h_{N2}\\ \cdots & \cdots & \cdots & \cdots \\ m_{1}h_{1N} & m_{2}h_{2N} & \cdots & 1 \end{array}\right)$$

and the diagonal matrix

(9) $$\tilde{E}=h\;\mathrm{diag}\left[m_{n},\;n=\bar{1,N}\right].$$

The vector $\tilde{F}\left(\mu ,z\right)\left[sh\right]$ consists of the components

(10) $${\tilde{f}}_{n}\left(z\left[sh\right]\right)=h\sum_{k=1}^{M}q_{kn}{\left(z\left[sh\right]\right)}^{c_{kn}},\;n=\bar{1,N},\;s=\bar{0,K-1}.$$

For each time sh, the variable *z* satisfies the equation

(11) $$\sum_{k=1}^{M}\sum_{n=1}^{N}c_{kn}q_{kn}{\left(z\left[sh\right]\right)}^{c_{kn}}=T\left[sh\right],\;s=\bar{0,K-1},$$

i.e., there exist *K* values $z=z^{*}\left[sh\right],\;s=\bar{0,K-1}$.

The randomized version of this model is described by Equations (7)–(11) but some parameters (variables) have random character. These are two randomized parameters, $b_{1}$ and $b_{2}$, as well as the variable $z=b_{3}$, all of the interval type. More specifically, the parameters $b_{1}$ and $b_{2}$ belong to the intervals

(12) $$B_{1}=\left[b_{1}^{-},b_{1}^{+}\right],\;B_{2}=\left[b_{2}^{-},b_{2}^{+}\right].$$

The interval $B_{3}$ of the variable $b_{3}$ is given by Equation (11).

**Theorem** **1.** *Let the parameters* $b_{kn}$ *and* $c_{kn}$ *in Equation (11) be positive and* $c_{kn}\in \left[0,1\right].$ *Then, the solution* $b_{3}^{*}$ *of this equation belongs to the interval*

(13) $$B_{3}=\left[b_{3}^{-},b_{3}^{+}\right],$$

*where*

(14) $$\begin{array}{ccc} b_{3}^{-} & = & {\left(\frac{T[sh]}{MNc_{max}b_{max}}\right)}^{1/c_{max}};\;b_{3}^{+}={\left(\frac{T[sh]}{MNc_{min}b_{min}}\right)}^{1/c_{min}};\\ c_{min} & = & \min_{kn}c_{kn},\;c_{max}=\max_{kn}c_{kn};\;b_{min}=\min_{kn}b_{kn},b_{max}=\max_{kn}b_{kn}. \end{array}$$

The proof is postponed to the Appendix A.

Therefore, the randomized dynamic model in Equations (7)–(11) includes three random parameters $b=\{b_{1},b_{2},b_{3}\}$ of the interval type that are defined over the three-dimensional cube with faces (Equations (12) and (13)), i.e.,

(15) $$B=\overset{3}{\underset{j=1}{⨂}}B_{j}.$$

The probabilistic properties of the randomized parameters are described by a continuously differentiable PDF W(b).

By assumption, real distributions of regional population sizes contain errors that are simulated by a random vector $\bar{\xi }\left[sh\right]\in R^{N}$ with the interval components

(16) $$\bar{\xi }\left[sh\right]\in \Xi_{s}=\left[{\bar{\xi }}^{-}\left[sh\right],{\bar{\xi }}^{+}\left[sh\right]\right].$$

The probabilistic properties of this vector are described by a continuously differentiable PDF $Q(\bar{\xi })$.

The measured output of the randomized model has an additive noise,

(17) $$v\left[sh\right]=K\left[sh\right]+\bar{\xi }\left[sh\right].$$

## 3. Characterization of Empirical Risk and Measurement Noises

*Construct a synthetic functional*$J[W\left(b\right),Q\left(\bar{\xi }\right)]$ that depends on the PDFs of the model parameters and measurement noises for assessing in quantitative terms the empirical risk (the difference between the regional population distribution generated by the model in Equations (7)–(11) and the real counterpart) and the guaranteed power of these noises. The functional must have components characterizing an intrinsic uncertainty of randomized machine learning (RML) procedures, the approximation quality of empirical balances (the empirical risk) and the worst properties of the corresponding random interval-type noises.

*1. Uncertainty.* In accordance with the general concept of RML, the first component among the listed ones is *an entropy functional* that describes the level of uncertainty:

(18) $$H\left[b\right),Q\left(\bar{\xi }\right)]=-\int_{B}W\left(b\right)\ln W\left(b\right)db-\int_{\Xi }Q\left(\bar{\xi }\right)\ln Q\left(\bar{\xi }\right)d\bar{\xi }.$$

The two other functional components are constructed using Hölder’s vector and matrix norms (The vector norm has the form ${∥a∥}_{\infty }=\max_{n}\left|a_{n}\right|$; the matrix norm, the form ${∥A∥}_{\infty }=\max_{ij}\left|a_{ij}\right|$.) [16].

*2. Approximate empirical balances.* First, consider a characterization of *the empirical risk*. For the model in Equations (7)–(11), the deviation between the output and real data vectors is given by

(19) $$\bar{\varepsilon }\left[sh\right]=\left(b_{1}b_{2}\tilde{A}-b_{1}\tilde{E}\right)Y\left[sh\right]+F\left(b_{3}\left[sh\right]\right)-Y\left[sh\right],\;s=\bar{0,K-1}.$$

Using well-known inequalities for the matrix and vector norms, it is possible to write

(20) $$\begin{array}{ccc} ∥\bar{\epsilon }{\left[sh\right]∥}_{\infty } & \leq & ∥\left(b_{1}b_{2}\tilde{A}-b_{1}\tilde{E}\right){∥}_{\infty }{∥Y\left[sh\right]∥}_{\infty }+∥F\left(b_{3}\left[sh\right]\right){∥}_{\infty }+{∥Y\left[\left(s+1\right)h\right]∥}_{\infty }=\\ & = & \varphi \left(b_{1},b_{2},b_{3},s\right),\;s=\bar{0,K-1}. \end{array}$$

Introducing the average matrix and vector norms over the observation interval,

(21) $$\begin{array}{cc} & \varphi \left(b_{1},b_{2},b_{3}\right)\leq h\left(\frac{1}{K}\sum_{s=0}^{K-1}\max_{n}y_{n}\left[sh\right]\right)\left(b_{1}\max_{n}m_{n}+b_{1}b_{2}\max_{i,j}h_{ij}\right)+\\ + & \frac{1}{K}\sum_{s=0}^{K-1}\max_{n}y_{n}\left[\left(s+1\right)h\right]+MNc_{max}b_{max}{\left(b_{3}\right)}^{c_{max}}. \end{array}$$

The parameters $b_{1}$ and $b_{2}$ take values within the intervals $B_{1}$ and $B_{2}$ (Equation (12)) while the parameter $b_{3}$ within the interval

(22) $$B_{3}=\left[{\left(\frac{T_{max}}{MNc_{max}q_{max}}\right)}^{1/c_{max}},{\left(\frac{T_{max}}{MNc_{min}q_{min}}\right)}^{1/c_{min}}\right],$$

where

(23) $$T_{max}=\max_{s}T\left[sh\right].$$

Denote

(24) $$\begin{array}{ccc} U_{1} & = & h\left(\frac{1}{K}\sum_{s=0}^{K-1}\max_{n}y_{n}\left[sh\right]\right)\max_{n}m_{n};\;U_{2}=h\left(\frac{1}{K}\sum_{s=0}^{K-1}\max_{n}y_{n}\left[sh\right]\right)\max_{i,j}h_{ij};\\ U_{3} & = & MNhc_{max}b_{max};\;U_{4}=\frac{1}{K}\sum_{s=0}^{K-1}\max_{n}y_{n}\left[\left(s+1\right)h\right]. \end{array}$$

Then, the function $\varphi (b_{1},b_{2},b_{3})$ takes the form

(25) $$\varphi \left(b_{1},b_{2},b_{3}\right)=b_{1}U_{1}+b_{1}b_{2}U_{2}+{\left(b_{3}\right)}^{c_{max}}U_{3}+U_{4}.$$

Note that the coefficients $U_{1},\ldots ,U_{4}$ are determined by real data on regional population distributions and also by the characteristics of internal migration within the system GFI and immigration flows from the system SL.

The equality in Equation (25) defines a function $\varphi (b_{1},b_{2},b_{3})$ of random variables. Let its expectation be the characteristic of the empirical risk, i.e.,

(26) $$r\left[W\left(b\right)\right]=\int_{B}W\left(b\right)\varphi \left(b\right)db,$$

where $B=B_{1}⊗B_{2}⊗B_{3}$ and the intervals $B_{1}$ and $B_{2}$ have given limits. At the same time, the limits of the interval $B_{3}$ are specified by the equalities in Equation (22).

*Power of noises.* The measurement noises are simulated by random vectors $\bar{\xi }\left[sh\right]\in R^{N},\;s=\bar{0,K-1}$. The components of these vectors may have different domains (ranges of values) at different times $s=\bar{0,K-1}$. For each time, introduce the Euclidean norm $∥\bar{\xi }{\left[sh\right]∥}_{N}^{2}$ and its expectation

(27) $$n_{s}\left[Q\left(\bar{\xi }\left[sh\right]\right)\right]=\int_{\Xi }Q\left(\bar{\xi }\left[sh\right]\right){∥\bar{\xi }\left[sh\right]∥}_{N}^{2}d\bar{\xi }\left[sh\right].$$

The average expectation of this norm over the time interval has the form

(28) $${\bar{n}}_{s}\left[Q\left(\bar{\xi }\left[sh\right]\right)\right]=\frac{1}{K}\sum_{s=0}^{K-1}n_{s}\left[Q\left(\bar{\xi }\left[sh\right]\right)\right].$$

If the measurement noises are the same on the observation interval, then the noise power functional can be written as

(29) $${\bar{n}}_{s}\left[Q\left(\bar{\xi }\left[sh\right]\right)\right]=n\left[Q\left(\bar{\xi }\right)\right]=\int_{\Xi }Q\left(\bar{\xi }\right){∥\bar{\xi }∥}_{N}^{2}d\bar{\xi }.$$

This formula involves the Euclidean norm for a quantitative characterization of the noise power. However, it is possible to choose other norms depending on problem specifics.

## 4. Soft Randomized Estimation of Model Parameters

The model characteristics and measurement noises are estimated using a learning data collection: the real cost of immigrants maintenance T[0],⋯,T[(K−1)h] (input data) and the real distributions of regional population sizes Y[0],⋯,Y[(K−1)h] (output data).

In accordance with the general procedure of soft randomized machine learning [15], the optimal probability density functions W(b) (model parameters) and $Q(\bar{\xi })$ (measurement noises) are calculated by the constrained minimization of the synthetic functional $J[W\left(b\right),Q\left(\bar{\xi }\right)]$ that contains the following functionals:

- the entropy
  (30) $$H\left[W\left(b\right)\right]=-\int_{B}W\left(b\right)\ln W\left(b\right)db-\int_{\Xi }Q\left(\bar{\xi }\right)\ln Q\left(\bar{\xi }\right)d\bar{\xi };$$
- the average empirical risk over the observation interval
  (31) $$r\left[W\left(b\right)\right]=\int_{B}W\left(b\right)\left(b_{1}U_{1}+b_{1}b_{2}U_{2}+{\left(b_{3}\right)}^{c_{max}}U_{3}+U_{4}\right)db;$$
  and
- the average error norm
  (32) $$n\left[Q\left(\bar{\xi }\right)\right]=\int_{\Xi }Q\left(\bar{\xi }\right)\sum_{i=1}^{N}\xi_{i}^{2}d\bar{\xi }.$$

The soft randomized learning algorithm has the form

(33) $$\begin{array}{c} J\left[W\left(b\right),Q\left(\bar{\xi }\right)\right]=H\left[W\left(b\right)\right]-r\left[W\left(b\right)\right]-n\left[Q\left(\bar{\xi }\right)\right]\Rightarrow \max,\\ \int_{B}W\left(b\right)db=1,\;\int_{\Xi }Q\left(\bar{\xi }\right)d\bar{\xi }=1. \end{array}$$

The solution of this problem is the optimal PDFs under maximal uncertainty, for the model parameters of the form

(34) $$W^{*}\left(b\right)=\frac{\exp\left(b_{1}U_{1}-b_{1}b_{2}U_{2}-{\left(b_{3}\right)}^{c_{max}}U_{3}-U_{4}\right)}{P},$$

where

(35) $$P=\int_{B}\exp\left(b_{1}U_{1}-b_{1}b_{2}U_{2}-{\left(b_{3}\right)}^{c_{max}}U_{3}-U_{4}\right)db,$$

and for the measurement noises of the form

(36) $$Q^{*}\left(\bar{\xi }\right)=\frac{\exp\left(-\sum_{i=1}^{N}\xi_{i}^{2}\right)}{Q},$$

where

(37) $$Q=\int_{\Xi }\exp\left(-\sum_{i=1}^{N}\xi_{i}^{2}\right)d\bar{\xi }.$$

In the case of soft randomization, there is no need for solving the empirical balance equations, which have high complexity and computational intensiveness due to the presence of integral components. Here, computational resources are required for the normalization procedure of the resulting PDFs. On the other hand, the morphology of the optimal PDFs depends on a specific choice of the approximate data balancing criterion and a numerical characterization of the measurement noises.

## 5. Randomized Forecasting of Dynamic Migratory Interaction

Consider randomized forecasting of dynamic migratory interaction using the principle of soft randomization. Let $T_{pr}=\left[s_{0}h,s_{pr}h\right]$ be the forecasting interval and assume the initial state (the regional population distribution at the initial time $s_{0}h$) coincides with the real distribution, i.e., $K\left[s_{0}h\right]=Y\left[s_{0}h\right]$. The shared cost of the system GFI to maintain immigrants is distributed in accordance with a given scenario. For each scenario, the value $T_{max}$ and also the interval $B_{3}$ in Equations (12), (22), and (23) are determined.

The forecasted trajectories are constructed using the randomized model in Equations (7), (10), and (11)

(38) $$\begin{array}{ccc} K[(s+1)h] & = & \left(b_{1}b_{2}\tilde{A}-b_{1}\tilde{E}\right)K\left[sh\right]+F\left[sh\;|\;b_{3}\right],\\ F[sh\;|\;b_{3}] & = & \{\sum_{k=1}^{M}b_{kn}{\left(b_{3}\right)}^{c_{kn}},\;n=\bar{1,N}\},\\ s & = & \bar{s_{0},s_{pr}},\;K\left[s_{0}h\right]=Y\left[s_{0}h\right]. \end{array}$$

The randomized parameters $b_{1},b_{2},$ and $b_{3}$ take values within the corresponding intervals with the probability density function $W^{*}\left(b\right)$ (Equation (34)).

An ensemble of the forecasted trajectories for the model’s output is obtained taking into account a random vector $\bar{\xi }\in \Xi$ with the PDF $Q^{*}\left(\bar{\xi }\right)$ (Equation (36)):

(39) $$v\left[sh\right]=K\left[sh\right]+\bar{\xi },\;s=\bar{s_{0},s_{pr}}.$$

For each scenario $T\left[s_{0}h\right],\cdots ,T\left[s_{pr}h\right],$ an ensemble K of random forecasting trajectories is generated via sampling (the transformation of a PDF into a corresponding sequence of random vectors of length *I*) of the optimal PDFs of the model parameters and measurement noises for each time sh. The resulting ensemble allows deriving empirical estimates of different numerical characteristics as follows:

- the average trajectory
  (40) $$\bar{K}\left[sh\right]=\frac{1}{I}\sum_{i=1}^{I}K^{\left(i\right)}\left[sh\right],\;s=\bar{s_{0},s_{pr}};$$
- the variance trajectory
  (41) $${\bar{\sigma }}^{2}\left[sh\right]=\frac{1}{I-1}\sum_{i=1}^{I}{∥K^{\left(i\right)}\left[sh\right]-\bar{K}\left[sh\right]∥}^{2},\;s=\bar{s_{0},s_{pr}};$$
- the variance pipe, i.e., the set of random trajectories that almost surely (since an ensemble consists of a finite number of trajectories, the matter concerns not probability but its empirical estimate) belong to the domain
  (42) $$D=\{K\left[sh\right]:\bar{K}\left[sh\right]-{\bar{\sigma }}^{2}\left[sh\right]\leq K\left[sh\right]\leq \bar{K}\left[sh\right]+{\bar{\sigma }}^{2}\left[sh\right],\;s=\bar{s_{0},s_{pr}}\};$$
- the empirical probability distribution and its dynamics on the forecasting interval
  (43) $$P\left(K\left[sh\right]\leq \Delta ,\;s=\bar{s_{0},s_{pr}}\right)=\frac{I_{\Delta }}{I},$$
  where $I_{\Delta }$ denotes the number of vectors K[sh] whose components are smaller than Δ; and
- the median trajectory $\hat{K}\left[sh\right],s=\bar{s_{0},s_{pr}}$, which satisfies the equation
  (44) $$P\left(K\left[sh\right]\right)=0,5;\;s=\bar{s_{0},s_{pr}}.$$

The ensemble K can be used to calculate other characteristics, e.g., α-quantiles, confidence probabilities, etc.

## 6. Example

The appearance of territories with low economic status always causes the growth of immigration. The early 2000s were remarkable for the formation of several such territories in Northern and Central Africa, the Near East, Afghanistan, etc. As a result, tens of millions of migrants moved to the EU as the level of life in these territories dropped below the subsistence minimum. The EU countries have to allocate considerable financial resources for their filtering and accommodation, which are often unacceptable. An example below illustrates the use of soft randomization for estimating and forecasting of immigration flows from Syria (1) and Libya (2) (the system SL) to Germany (1), France (2), and Italy (3) (the system GFI).

*1. Randomized model, parameters, measurement errors, time intervals, and real data collections.* Choose the randomized mathematical model (Equation (25)) with the normalized variables

(45) $$p_{n}\left[sh\right]=\frac{K_{n}\left[sh\right]}{K_{max}},\;n=\bar{1,3}.$$

This gives

(46) $$\begin{array}{ccc} p_{n}\left[\left(s+1\right)h\right] & = & \left(1-b_{1}m_{n}\right)p_{n}\left[sh\right]+hb_{1}b_{2}\sum_{i=1,i\neq n}^{3}m_{i}h_{in}p_{i}\left[sh\right]+hf_{n}\left[sh\right],\\ f_{n}\left[sh\right] & = & \sum_{i=1}^{M}b_{in}b_{3}^{c_{in}},\;n=\bar{1,3},\\ T[sh] & = & \sum_{n=1}^{3}\sum_{i=1}^{2}c_{in}b_{in}b_{3}^{c_{in}}. \end{array}$$

The state variables of the system GFI and also the immigration flows from the system SL are normalized, i.e.,

(47) $$0\leq p_{n}\left[sh\right]\leq 1,\;0\leq f_{n}\left[sh\right]\leq 1,\;n=\bar{1,N}.$$

The variable $z^{*}$ characterizes the entropy operator of the immigration process and satisfies the last equation in Equation (46). The values of the parameters $m_{i},h_{in},b_{in},$ and $c_{in}$ are combined in Table 1, where columns are different values of corresponding parameter. Recall that the two lowest rows of Table 1 indicate the values of the parameters $c_{in}$. By assumption, the regions of both systems have the same specific cost.

In accordance with this table, $m_{max}=0.5,h_{max}=0.5,b_{min}=0.3,b_{max}=0.4,$ and $c_{max}=c_{min}=c=0.5$. The measurement errors of population sizes $\bar{\xi }\left[sh\right]\in R^{3}$ (in normalized units) belong to the intervals

(48) $$\bar{\xi }\left[sh\right]\in \Xi =\left[{\bar{\xi }}_{-},{\bar{\xi }}_{+}\right],\;\xi_{n}^{\pm }=0.01,$$

and by assumption they have the same limits for times sh.

The normalized observation (model output) has the form

(49) $$v\left[sh\right]=p\left[sh\right]+\bar{\xi }\left[sh\right].$$

The random parameter model in Equation (46) was employed for estimating parameter characteristics and testing on corresponding time intervals with step h=1 *year*:

- $T_{est}=2009$–2013 as the estimation interval; and
- $T_{tst}=2014$–2018 as the testing interval.

*2. Entropy estimation of PDFs of model parameters and measurement noises (interval*$T_{est}$*).* This problem was solved using available data on regional population distribution for Germany (n=1), France (n=2), and Italy (n=3) and also on the shared cost of immigrants maintenance on the estimation interval (see Table 2 and UNdata service at https://data.un.org/).

In this model, the random parameters $b_{1},b_{2},$ and $b_{3}$ take values within the intervals

(50) $$b_{1}\in B_{1}=\left[1.0,2.5\right];\;b_{2}\in B_{2}=\left[0.5,1.8\right],\;b_{3}\in B_{3}=\left[0.3,1.5\right].$$

In accordance with Equation (24),

(51) $$U_{1}=0.5;\;U_{2}=0.5;\;U_{3}=1.2;\;U_{4}=0.986.$$

Then, the soft RML procedure yields the following optimal PDFs of the model parameters and measurement noises:

(52) $$\begin{array}{ccc} W^{*}\left(b\right) & = & \frac{\exp\left(-0.5b_{1}-0.5b_{1}b_{2}-1.2b_{3}^{0.5}-0.986\right)}{W},\\ Q^{*}\left(\bar{\xi }\right) & = & \frac{\exp\left(-\sum_{n=1}^{3}\xi_{n}^{2}\right)}{Q}, \end{array}$$

where

(53) $$\begin{array}{ccc} W & = & \int_{B_{1}}\;\int_{B_{2}}\;\int_{B_{3}}\exp\left(-0.5b_{1}-0.5b_{1}b_{2}-1.2b_{3}^{0.5}-0.986\right)db_{1}db_{2}db_{3},\\ Q & = & \prod_{n=1}^{3}\int_{-0.01}^{0.01}\exp\left(-\xi^{2}\right)d\xi . \end{array}$$

**Figure 1:**
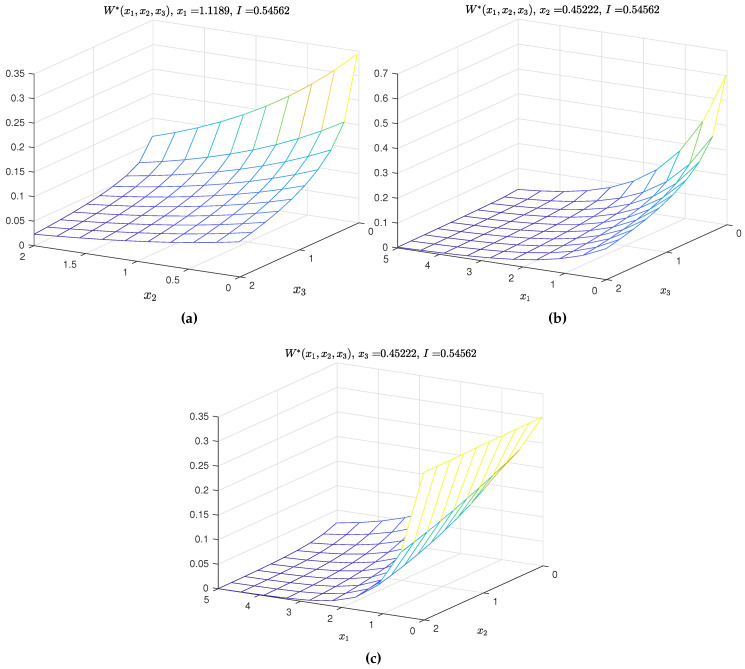
2-dimensional section of *W*.

**Figure 2:**
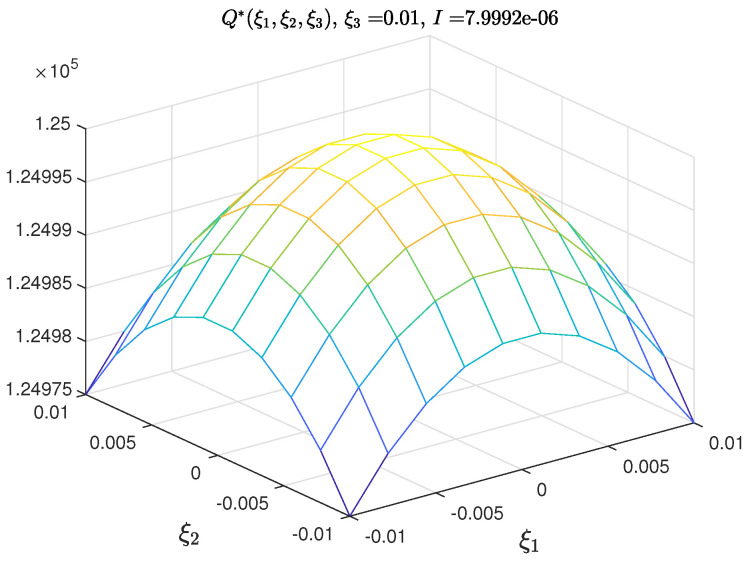
2-dimensional section of *Q*.

The two-dimensional sections of the three-dimensional PDFs of the model parameters are shown in Figure 1a–c, while the graphs of the PDFs of the measurementnoises in Figure 2.

*3. Model testing.* The randomized model in Equation (49) with the optimal PDFs in Equations (52) and (53) was tested using the above data on regional population sizes from the UNdata service (https://data.un.org/) (see Table 3). This table also presents the testing results in terms of the ensemble-average trajectories ${\bar{p}}_{1}\left[sh\right],{\bar{p}}_{2}\left[sh\right],$ and ${\bar{p}}_{3}\left[sh\right]$.

**Figure 3:**
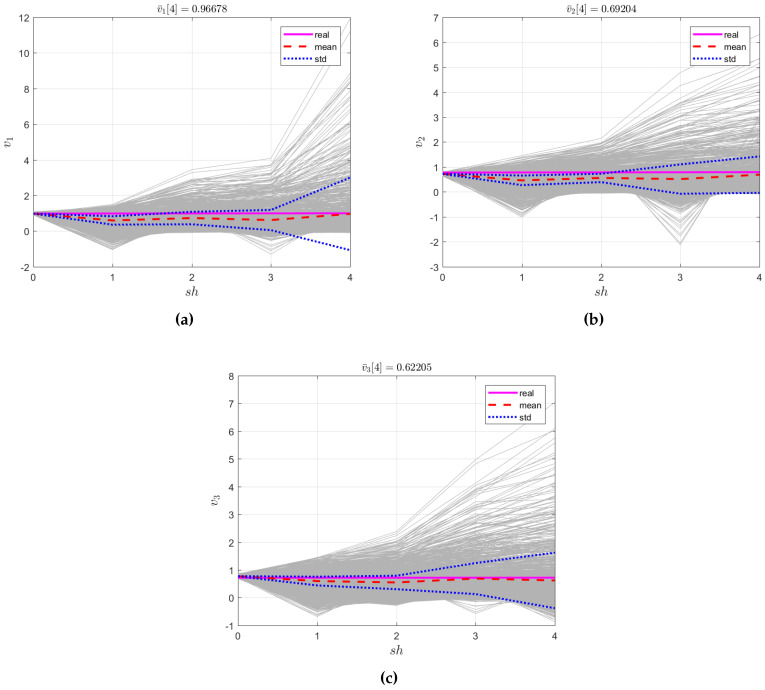
(**a**) ${\bar{v}}_{1}$ [4], (**b**) ${\bar{v}}_{2}$ [4], (**c**) ${\bar{v}}_{3}$ [4].

Testing was performed via sampling of the randomized interval parameters with the PDFs in Equations (52) and (53) and construction of the corresponding trajectories by Equation (49). Figure 3a–c shows ensembles of such trajectories $v_{1}\left[sh\right],v_{2}\left[sh\right],v\left[sh\right]$ as well as the ensemble-average trajectories ${\bar{v}}_{1}\left[sh\right],{\bar{v}}_{2}\left[sh\right],{\bar{v}}_{3}\left[sh\right]$ (Graph 1); the real trajectories $y_{1}\left[sh\right],y_{2}\left[sh\right],y_{3}\left[sh\right]$ of regional population sizes (Graph 2); and the limits of the variance pipes ${\bar{p}}_{1}^{*}\left[sh\right]\pm \sigma_{1},{\bar{p}}_{2}^{*}\left[sh\right]\pm \sigma_{2},{\bar{p}}_{3}^{*}\left[sh\right]\pm \sigma_{3}$ (Graph 3).

The testing accuracy was estimated in terms of the relative root-mean-square error

(54) $$\delta_{n}=\frac{\sqrt{\sum_{s=0}^{4}{\left({\bar{p}}_{n}\left[sh\right]-y_{n}\left[sh\right]\right)}^{2}}}{\sqrt{\sum_{s=0}^{4}{\left({\bar{p}}_{n}\left[sh\right]\right)}^{2}}+\sqrt{\sum_{s=0}^{4}{\left(y_{n}\left[sh\right]\right)}^{2}}}.$$

In the example under study, it constituted 4.6% (Region 1), 3.5% (Region 2), and 2.6% (Region 3).

## 7. Conclusions

This paper has developed a mathematical model for dynamic migratory interaction of regional systems with locally stationary states described by corresponding entropy operators. The model incorporates random parameters, and their probabilistic characteristics—the probability density functions of system parameters and measurement noises—have been calculated using soft randomized machine learning. An example of migratory interaction modeling and testing has been given.

## Figures and Tables

**Table 1:** Values of relative parameters.

$m_{n}$	0.43	0.50	0.40
$h_{1n}$	0	0.3	0.3
$h_{2n}$	0.3	0	0.3
$h_{3n}$	0.5	0.4	0
$b_{1n}$	0.4	0.3	0.3
$b_{2n}$	0.3	0.1	0.4
$c_{1n}$	0.4	0.4	0.3
$c_{2n}$	0.4	0.4	0.3

**Table 2:** Input and output data collections.

Year	2009	2010	2011	2012	2013
*s*	0	1	2	3	4
$Y_{1}\left[s\right]$	81.90	81.77	80.27	80.42	80.64
$y_{1}\left[s\right]$	1.00	0.998	0.980	0.982	0.985
$Y_{2}\left[s\right]$	62.47	62.80	63.11	63.41	63.70
$y_{2}\left[s\right]$	0.762	0.767	0.771	0.774	0.778
$Y_{3}\left[s\right]$	59.39	59.53	59.63	59.71	59.75
$y_{3}\left[s\right]$	0.725	0.727	0.728	0.729	0.726
T[s] (*billion*)	0.093	0.094	0.095	0.096	0.097

**Table 3:** Input and output data collections.

Year	2014	2015	2016	2017	2018
*s*	0	1	2	3	4
$Y_{1}\left[s\right]$	81.489	81.707	82.063	82.386	82.674
$y_{1}\left[s\right]$	0.985	0.988	0.993	0.996	1.000
${\bar{p}}_{1}\left[sh\right]$	0.986	0.615	0.743	0.639	0.999
$Y_{2}\left[s\right]$	64.190	64.457	64.791	65.134	65.484
$y_{2}\left[s\right]$	0.721	0.472	0.564	0.529	0.708
${\bar{p}}_{2}\left[sh\right]$	0.722	0.695	0.707	0.691	0.715
$Y_{3}\left[s\right]$	59.585	59.504	59.504	59.509	59.516
$y_{3}\left[s\right]$	0.775	0.609	0.562	0.699	0.650
${\bar{p}}_{3}\left[sh\right]$	0.776	0.617	0.607	0.705	0.628
T[s] (*billion*)	0.097	0.097	0.097	0.098	0.098

## Appendix A

**Proof** **of Theorem 1.** Consider the function

(A1) $$\varphi \left(z\right)=\mu \sum_{k=1}^{M}\sum_{n=1}^{N}c_{kn}q_{kn}{\left(z\left[sh\right]\right)}^{c_{kn}},$$

which appears on the left-hand side of Equation (19). Taking advantage of the obvious inequalities,

(A2) $$\varphi_{-}\left(z\right)=MN\mu c_{min}q_{min}{\left(z\right)}^{c_{min}}<\varphi \left(z\right)<MN\mu c_{max}q_{max}{\left(z\right)}^{c_{max}}=\varphi_{+}\left(z\right).$$

The variables are $0<c_{min}<1,\;0<c_{max}<1,\;c_{min}<c_{max}$, and $c_{min}<c_{kn}<c_{max}$. Consider the equations

(A3) $$\varphi_{-}\left(z\right)=T\left[sh\right],\;\varphi \left(z\right)=T\left[sh\right],\;\varphi_{+}\left(z\right)=T\left[sh\right].$$

The functions $\varphi_{-}\left(z\right),\varphi \left(z\right),$ and $\varphi_{+}\left(z\right)$ are strictly convex. Therefore, the solutions of these equations has the relationship

(A4) $$z_{-}<z^{*}<z_{+},$$

which concludes the proof of Theorem 1. □
